# Evaluation of Safety and Effectiveness of Elvitegravir/Cobicistat/Emtricitabine/Tenofovir Alafenamide Switch Followed by Ledipasvir/Sofosbuvir HCV Therapy in HIV–HCV Coinfection

**DOI:** 10.1093/ofid/ofz318

**Published:** 2019-07-03

**Authors:** Mary-Anne Doyle, Terry Lee, Joel Singer, Angela Crawley, Marina Klein, Curtis Cooper

**Affiliations:** 1 Division of Endocrinology and Metabolism, Department of Medicine, University of Ottawa, Ottawa, Ontario, Canada; 2 Ottawa Hospital Research Institute, Ottawa, Ontario, Canada; 3 CIHR Canadian HIV Trials Network, School of Population and Public Health, University of British Columbia, Vancouver, British Columbia, Canada; 4 Division of Infectious Diseases, Department of Medicine, University of Ottawa, Ottawa, Ontario, Canada; 5 Department of Medicine, McGill University, Montreal, Quebec, Canada

**Keywords:** viral hepatitis, cirrhosis, antiviral therapy, insulin resistance, lipid metabolism

## Abstract

**Background:**

We conducted a pilot study assessing the feasibility, efficacy, and safety of a simplified combination HIV antiretroviral and hepatitis C virus (HCV) antiviral regimen in HIV–HCV coinfection.

**Methods:**

Participants on suppressive antiretrovirals and HCV genotype 1 infection were switched to single-tablet daily-dosed elvitegravir/cobicistat/emtricitabine/tenofovir alafenamide (E/C/F/TAF) and 1 month later initiated single-tablet-regimen daily-dosed ledipasvir-sofosbuvir for 12 weeks. E/C/F/TAF was continued during HCV treatment and for 12 weeks after.

**Results:**

Twenty-six individuals were screened, 25 enrolled, and 23 completed all HIV and HCV treatment. Participants were predominantly male, with a mean age (SD) of 55 (7.5) years. The median transient elastography score (interquartile range [IQR]) was 5.9 (5.3 to 7.6) kPa, and the mean CD4 count (SD) was 579 (223) cells/µL. The median adherence to HCV medications, assessed by pill count, was 100% (95% confidence interval [CI], 100%–100%), and HIV ranged from 99% to 100% (100%; 95% CI, 90%–100%) over the 7-month study duration. HIV undetectability was maintained in all but 1 participant enrolled with unsuspected multiclass resistance. Treatment was well tolerated, with no study medication modification due to adverse events and no serious adverse event related to the study drug. All participants achieved sustained virological response. The mean CD4 count (SD) increased to 673 (361) cells/µL, and the fibrosis score (IQR) declined to 5.2 (4.4 to 7.4) kPa by week 12 after HCV treatment. There was no treatment effect on glucose metabolism. Cholesterol increased during and after treatment.

**Conclusions:**

Provision of this 2-tablet daily HIV–HCV regimen is feasible, well tolerated, and safe, avoids drug–drug interactions between HIV and HCV medications, maintains HIV suppression in the absence of drug resistance, and is highly curative of HCV.

Chronic hepatitis C affects 1% of the world’s population and is a leading cause of cirrhosis, hepatocellular carcinoma, and liver transplantation [1, 2]. More than 350 000 deaths per year are attributable to hepatitis C virus (HCV)–related complications [1]. As a consequence of shared risk factors for exposure and routes of transmission, HIV and HCV are often found concurrently [3]. Globally it is estimated that approximately 6.2% of HIV-infected individuals (2.3 million) are coinfected with HCV [4].

Individuals with HIV–HCV coinfection tend to have higher HCV viral loads, are less likely to respond to interferon-based HCV antiviral treatment, and are at higher risk for progression of liver disease, cirrhosis, and end-stage liver disease [5–7]. As such, liver disease has historically been a leading cause of morbidity and mortality in HIV–HCV-coinfected patients [8]. In both HCV mono-infection and HIV–HCV coinfection, HCV treatment and viral clearance are associated with improved outcomes and a reduction in morbidity and mortality. Until recently, HCV treatment has consisted of interferon-based regimens, which are associated with multiple side effects, have interactions with HIV antiretrovirals, have complicated dosing schedules, and are less efficacious in HIV-coinfected patients.

Treatment for HCV has evolved in recent years with the emergence of interferon-free direct-acting antivirals (DAAs). These new treatments are shorter in duration, have improved safety profiles, are better tolerated, and have higher sustained virological response (SVR) rates [9–11]. Current standard-of-care DAA regimens also have fewer drug–drug interactions and present an opportunity to improve outcomes and HCV treatment success in this HIV–HCV patient population.

The availability of interferon-free HCV DAA antiviral therapy such as ledipasvir/sofosbuvir (LDV/SOF) allows for broad provision of treatment for populations living with HIV–HCV. With this is mind, the optimal management of antiretroviral therapy before initiating LDV/SOF treatment has not been established. Elvitegravir/cobicistat/emtricitabine/tenofovir alafenamide (E/C/F/TAF) is formulated as a single tablet, which facilitates adherence by once-daily dosing and reduced pill count. It is established to be effective at achieving and maintaining HIV virologic suppression [12, 13]. The safety profile of this HIV regimen is excellent. This formulation has been evaluated in HIV-infected patients and found to be of similar efficacy to other standard-of-care regimens and to have improved renal and bone safety compared with TDF-based treatment. Drug–drug interactions between HIV antiretrovirals and HCV antivirals remain a key obstacle to safe and effective delivery. The drug–drug interactions between E/C/F/TAF and LDV/SOF has been well evaluated, and no clinically significant interactions have been identified [14, 15]. Concerns about elevated tenofovir exposure with ledipasvir and cobicistat coadministration are thought to be diminished with use of the TAF formulation [16, 17]. A switch to E/C/F/TAF in the context of LDV/SOF HCV antiviral treatment preparation may be particularly beneficial because of its favorable side effect profile, once-daily single-tablet regimen formulation, known drug–drug interaction profile with LDV/SOF, neutral effect on liver fibrosis, and improved kidney and bone safety profile with the use of TAF. There are well-documented concerns related to the metabolic complications of certain HIV antiretrovirals, the metabolic effects of HIV and HCV infections themselves, and the lack of metabolic safety information related to HCV antivirals [18–24]. It is plausible that a switch to the metabolically inert E/C/F/TAF regimen plus HCV clearance may represent another benefit of this proposed HIV–HCV treatment strategy.

The objective of this study was to assess the feasibility, safety, and efficacy of switching HIV–HCV-infected individuals with suppressed HIV RNA to E/C/F/TAF followed by 12-week HCV antiviral treatment with LDV/SOF. Furthermore, as secondary outcomes, we assessed the effects of HCV clearance and viral cure on measures of liver fibrosis, immune function, metabolic parameters, and key patient-reported outcomes.

## METHODS

Approval for this study was obtained from the Ottawa Health Science Network Research Ethics Board and McGill University Health Centre Research Ethics Board; the study was registered with ClinicalTrials.gov (NCT02660905).

Participants for this open-label pilot study were recruited from The Ottawa Hospital Viral Hepatitis Program (Ottawa, Ontario) and McGill University Health Centre (Montreal, Quebec) between May 2016 and March 2017. All participants were 18 years or older, HCV genotype 1–infected, HCV RNA–positive for at least 6 months, documented with HIV <50 copies/mL on combination antiretroviral therapy for at least 12 weeks, and provided signed informed consent. Exclusion criteria included decompensated liver disease, use of drugs with contraindications or interactions with E/C/F/TAF and LDV/SOF, history of HIV integrase inhibitor resistance mutations, and platelets <50 ×10^9^/L. Participants who met the inclusion criteria were switched from their current antiretroviral regimen to E/C/F/TAF. Four weeks thereafter, participants initiated a 12-week course of LDV/SOF while continuing E/C/F/TAF.

Participant demographics, medical history, HIV RNA, CD4 count, HCV results (HCV RNA level, genotype), HBV serology, mode of HIV and HCV infection, length of time since infection diagnosis, and risk factors were collected at screening. Information on preexisting diabetes, the use of glucose and lipid-lowering medications, other concurrent liver disorders, estimates of alcohol consumption, smoking, history of illicit drug use, chronic kidney disease, transplant history, and immune suppressant was collected, as these are factors known to influence immune function, metabolic parameters, and progression of liver fibrosis.

Blood samples were collected for HCV RNA at screening, day 0 of E/C/F/TAF initiation (4 weeks before LDV/SOF dosing); day 0, week 4, and week 12 of LDV/SOF dosing; and 12 weeks after LDV/SOF dosing. Fasting insulin, glucose, total cholesterol, high-density lipoprotein cholesterol (HDL-C), low-density lipoprotein cholesterol (LDL-C)–triglycerides (TG), HbA1c, ApoA1, ApoA2, ApoB, ApoC2, ApoC3, and Apo E were measured at these same time points. Patients were advised to fast before blood draws. Homeostatic Model Assessment–Insulin Resistance (HOMA-IR) score was calculated as per the following: (glucose × insulin)/22.5. IR was defined as having a HOMA-IR >2. A cutoff of 2 was selected as this has been the cutoff point used in other studies assessing IR in HCV patients [25].

Liver fibrosis was determined by transient elastography (FibroScan) at screening, week 12 of LDV/SOF dosing, and 12 weeks after HCV treatment. A FibroScan F4 score >12.5 kPa was defined as cirrhotic. Controlled attenuation parameter (CAP) was utilized as a measure of steatosis and/or inflammation in the parenchyma of the liver.

Participants additionally completed the following questionnaires at weeks –4, 0, and 12 and 12 weeks post: Alcohol and Illicit Drug Use, International Physical Activity Questionnaire (IPAQ), and EuroQoL Group-5 Dimensional (EQ-5D-5L). The Alcohol Use Disorders Identification Test- Consumption (AUDIT-C) and in-house drug use questionnaires were used to evaluate alcohol consumption and illicit drug use. The IPAQ is a validated questionnaire that quantifies total physical activity during the last 7 days across 4 domains: leisure, domestic, work, and transportation-related activity. A total physical activity score was computed for sedentary, walking, moderate- and vigorous-intensity activities using type, frequency, duration, and metabolic equivalent of task (MET) for activities within each domain; it was reported as MET minutes per week. The IPAQ–Short Form also classifies total combined weekly physical activity scores as representing high, moderate, or low activity. The EQ-5D-5L is a quality of life assessment tool that evaluates health in 5 domains: Mobility, Self-care, Usual Activities, Pain/Discomfort, and Anxiety or Depression. A composite utility score is then generated.

A convenience sample of 25 was chosen for this pilot study. Given the sample size, if 100% SVR were attained, the lower bound of the 95% confidence interval (CI) would be 86.3%. Demographic and other baseline variables were summarized using percentage, mean, median, standard deviation, and interquartile range, as appropriate. The primary end point for this study was the feasibility of this HIV–HCV treatment, as determined by the proportion of patients who agreed to switch from their current ARV regimen and were screened for this study. Screen failures due to drug–drug interactions and the proportion of subjects who maintained >95% adherence to HIV and HCV antiviral therapies were evaluated as key markers of feasibility. Median adherence was assessed at week –4, week 0, and week 12 of LDV/SOF dosing, as well as 12 weeks after HCV treatment. Adherence was defined as the number of pills taken divided by the number of pills expected to be taken.

As secondary end points, we evaluated the proportion of participants achieving SVR12, the proportion maintaining undetectable HIV RNA levels, and the proportion of subjects discontinuing medications due to adverse events. In addition, changes in HOMA-IR, lipid levels, apolipoprotein parameters, and liver fibrosis scores were assessed from baseline to week 4, week 12, and 12 weeks after HCV treatment using the *t* test, Wilcoxon signed-rank test, or McNemar’s test, as appropriate. Adverse events were summarized by grade and relationship to treatment. Distribution-free confidence interval for percentile was based on the order statistics method, whereas exact confidence intervals for proportions were based on binomial distribution. Analyses were conducted using SAS 9.4 (SAS Institute, Cary, NC).

## RESULTS

**Figure 1.**
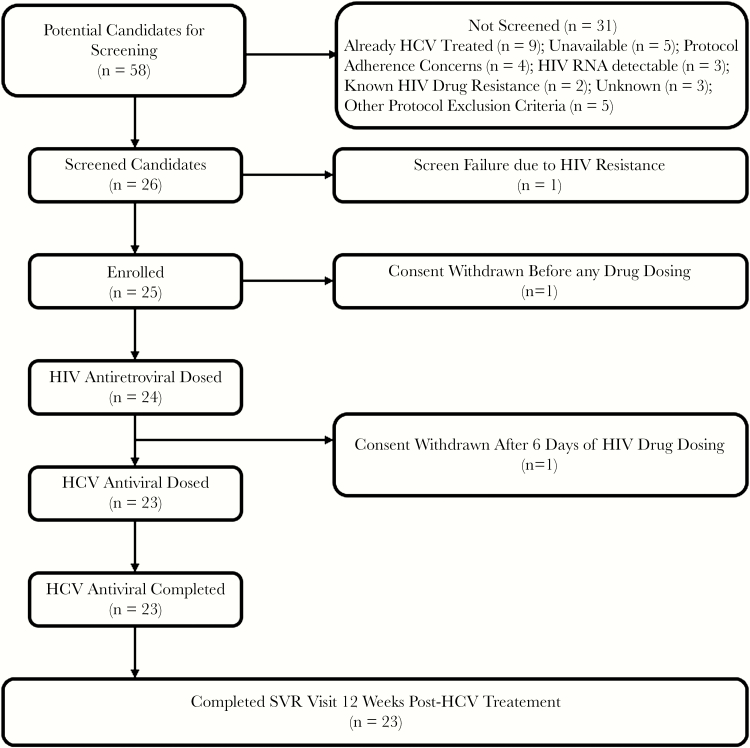
Flow diagram of participant screening, dosing, and protocol completion. Abbreviations: HCV, hepatitis C virus; SVR, sustained virological response.

Of 58 patients who were identified as potential study candidates, 26 were screened (44.8%) (Figure 1). Potential participants (n = 32) were not screened for a variety of reasons, the most common including having been already treated for HCV (n = 8), patient unavailability (n = 5), concerns related to protocol adherence (n = 4), and HIV RNA detectability (n = 3). There were no screen failures due to drug–drug interactions or prior antiretroviral resistance. Twenty-five participants were enrolled in this study. One participant withdrew consent before dosing. One participant withdrew consent and discontinued E/C/F/TAF after 6 days of doing. Twenty-three participants were switched to single-tablet daily-dosed E/C/F/TAF and completed LDV/SOF treatment for HCV.

Baseline characteristics are outlined in Table 1. Participants were predominantly male, with a mean age (SD) of 55 (7.5) years and a mean BMI (SD) of 23.9 (3.0) kg/m^2^. The most common mode of HIV and HCV infection acquisition was former injection drug use (76%), although in some cases more than 1 risk factor was identified in a specific participant. As per inclusion criteria, all participants were on antiretroviral regimens with fully suppressed HIV RNA levels for a minimum of 12 weeks. No participant had a history of documented or suspected integrase inhibitor resistance. Pre-E/C/F/TAF switch regimens included integrase inhibitor (n = 6, of which 3 were on a single-tablet regimen, 1 of which was boosted), protease inhibitor (n = 10), non-nucleoside reverse transcriptase inhibitor (n = 8), and all nucleoside-based regimens (n = 1). Most participants were infected with genotype 1a, with a mean HCV RNA level of 6.33 ×10^6^ IU/mL and a median transient elastography score (IQR) of 5.9 (5.3 to 7.6) kPa. Three patients had cirrhosis based on a stiffness score of >12.5 kPa. The mean CD4 cell count (SD) was 579 (223) cells/µL at baseline.

**Table 1.** Baseline Characteristics of Study Participants

Variable	All (n = 25)
Age, mean (SD), y	55.2 (7.5)
Male, No. (%)	24 (96.0)
Weight, mean (SD), kg	71.9 (11.3)
BMI,^b^ mean (SD), kg/m^2^	23.9 (3.0)
Race, No. (%)	
Indigenous	1 (4.0)
Asian	1 (4.0)
Black	1 (4.0)
Caucasian	19 (76.0)
Hispanic	1 (4.0)
Other^b^	2 (8.0)
Immigrant, No. (%)	4 (16.0)
Years since first HCV-positive test,^d^ mean (SD)	14.9 (7.5)
Mode of HCV acquisition, No. (%)^a^	
Sexual contact	5 (20.0)
Injection drug use	19 (76.0)
Occupational exposure	1 (4.0)
Unknown	3 (12.0)
HCV-RNA result, mean (SD), log10 IU/mL	6.33 (0.43)
HCV subtype, No. (%)	
1 (subtype unknown)	3 (12.0)
1a	19 (76.0)
1b	3 (12.0)
CD4 count, mean (SD), cells/µL	579.1 (222.6)
Years since first HIV-positive test,^d^ mean (SD), y	20.0 (5.3)
Mode of HIV acquisition, No. (%)^a^	
Sexual contact	9 (36.0)
Injection drug use	19 (76.0)
Blood product	0 (0.0)
Occupational exposure	1 (4.0)
Fasting glucose, median (IQR), mmol/L	4.80 (4.50 to 5.10)
Fasting insulin, median (IQR), pmol/L	54.10 (36.90 to 112.25)
HOMA-IR, median (IQR)	1.75 (1.15 to 3.14)
HbA1c, median (IQR), %	5.30 (5.20 to 5.50)
Total cholesterol, median (IQR), mmol/L	3.93 (3.24 to 4.50)
LDL, median (IQR), mmol/L	2.00 (1.70 to 2.41)
HDL, median (IQR), mmol/L	1.32 (1.07 to 1.59)
Triglycerides, median (IQR), mmol/L	0.99 (0.83 to 2.06)
FibroScan Stiffness >12.5 kPa, No. (%)	3 (12.0)
FibroScan CAP score, median (IQR)	233.0 (189.0 to 246.0)
ALT, median (IQR), U/L	54.0 (37.0 to 78.0)
AST, median (IQR), U/L	44.0 (29.0 to 54.0)
AUDIT-C, median (IQR)	2.0 (1.0 to 4.0)
Has problems with EQ-5D dimension, No. (%)^a,e^	
Mobility	8/23 (34.8)
Self-care	2/23 (8.7)
Usual Activities	10/23 (43.5)
Pain/Discomfort	19/23 (82.6)
Anxiety/Depression	15/23 (65.2)
EQ-5D: Your Health Today, median (IQR)	75.0 (70.0 to 85.0)
IPAQ-total MET-min/wk, median (IQR)	3915.0 (1558.5 to 6527.3)

Abbreviations: ALT, alanine aminotransferase; AST, aspartate aminitransferase; BMI, body mass index; CAP, controlled attenuation parameter; EQ-5D, EuroQoL Group–5 Dimensional; IPAQ, International Physical Activity Questionnaire; HCV, hepatitis C virus; HDL, high-density lipoprotein; HOMA-IR, Homeostatic Model Assessment–Insulin Resistance; IQR, interquartile range; LDL, low-density lipoprotein; MET, metabolic equivalent of task.

^a^Multiple factors can be selected for each patient.

^b^Height was missing for 2 patients.

^c^Burmese (n = 1) and East Asian (n = 1).

^d^If date of first positive test was unknown, date of HCV/HIV acquisition was used.

^e^Those who scored level 2 or higher were considered to have experienced problems.

Median adherence to HCV medications was 100% (95% CI, 100%–100%), and HIV medications ranged from 99% to 100% (100%; 95% CI, 90%–100%) over the 28-week study duration (Table 2). The proportion of participants maintaining >95% adherence to HCV medication was 94% (95% CI, 70%–100%) at week 12, and the proportion maintaining >95% adherence to HIV medications was 87% (95% CI, 60%–98%) at week 12. However, this proportion for >95% HIV medication adherence declined to 64% (95% CI, 35%–87%) at week 12 after HCV treatment.

**Table 2.** Adherence to HIV and HCV Treatments at Baseline and Throughout the Study

	Visit			
	Baseline	Week 4^a^	Week 12^a^	12 Weeks Post^a^
HIV drug compliance, No.^b^	17	16	15	14
Median (IQR)	100.0 (96.0 to 100.0)	100.0 (100.0 to 100.0)	100.0 (96.4 to 100.0)	99.0 (90.0 to 100.0)
HIV drug adherence >80%, No. (%)	16/17 (94.1)	16/16 (100.0)	15/15 (100.0)	13/14 (92.9)
HIV drug adherence >90%, No. (%)	16/17 (94.1)	16/16 (100.0)	15/15 (100.0)	10/14 (71.4)
HIV drug adherence >95%, No. (%)	14/17 (82.4)	13/16 (81.3)	13/15 (86.7)	9/14 (64.3)
HCV drug compliance, No.^b^		18	16	
Median (IQR)		100.0 (100.0 to 100.0)	100.0 (99.1 to 100.0)	
HCV drug adherence >80%, No. (%)		18/18 (100.0)	16/16 (100.0)	
HCV drug adherence >90%, No. (%)		17/18 (94.4)	15/16 (93.8)	
HCV drug adherence >95%, No. (%)		15/18 (83.3)	15/16 (93.8)	

Abbreviations: HCV, hepatitis C virus; IQR, interquartile range.

^a^Time points in relation to LDV/SOF dosing.

^b^Variable Nos. reflect missing drug adherence data at individual time points.

**Figure 2.**
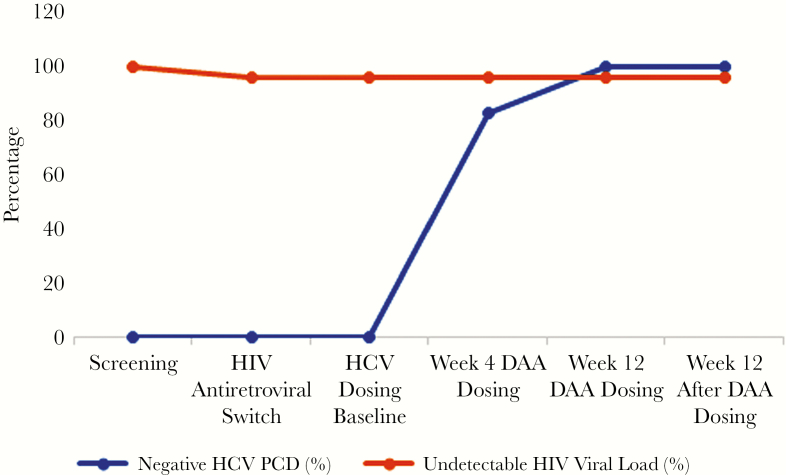
Percentage of patients with participants with negative HCV RNA and an undetectable HIV viral load at screening, baseline, and throughout the study period. Abbreviations: DAA, direct-acting antivirals; HCV, hepatitis C virus; PCR, polymerase chain reaction.

Nineteen participants (83%) cleared HCV RNA by week 4 of LPV/SOF treatment, and all participants cleared the virus by week 12 (Figure 2). All achieved SVR (100%; 95% CI, 85%–100%). HIV undetectability was maintained in 95% (95% CI, 78%–100%) of participants over the course of observation (Figure 2). The mean CD4 cell count (SD) increased to 673 (361) cells/ µL (*P* = .03), and the median transient elastography score (IQR) declined to 5.2 (4.4 to 7.4) kPa (*P* < .001) by week 12 after HCV treatment (Table 3).

**Table 3.** Change in Baseline Characteristics and Metabolic Parameters at Week 4, Week 12, and 12 Weeks Post-Treatment

		Change Relative to Baseline^a^		
Variable		Week 4	Week 12	Week 12 Post
FibroScan Stiffness	Median (IQR)		–0.30 (–2.90 to 0.50)	–1.40 (–2.00 to –0.40)
	*P*		.109	<.001
FibroScan CAP score	Median (IQR)		9.0 (–6.0 to 36.0)	4.0 (–21.0 to 20.0)
	*P*		.092	1.000
ALT	Median (IQR)	–21.0 (–54.0 to –8.0)	–26.0 (–72.0 to –9.0)	–25.5 (–52.0 to –12.0)
	*P*	<.001	<.001	<.001
AST	Median (IQR)	–14.0 (–52.0 to –2.0)	–16.0 (–47.0 to –5.0)	–19.0 (–42.0 to –2.0)
	*P*	<.001	<.001	<.001
CD4 count	Mean (SD)	33.2 (189.2)	55.9 (120.6)	105.7 (224.3)
	*P*	.055	.027	.042
Total cholesterol^b^	Median (IQR)	0.82 (0.59 to 1.15)	1.03 (0.22 to 1.65)	0.40 (–0.10 to 0.90)
	*P*	<.001	<.001	.022
LDL^b^	Median (IQR)	0.95 (0.40 to 1.20)	0.93 (0.40 to 1.45)	0.50 (0.10 to 0.90)
	*P*	.002	<.001	.001
HDL^b^	Median (IQR)	–0.07 (–0.19 to 0.28)	0.20 (0.01 to 0.29)	–0.02 (–0.21 to 0.19)
	*P*	.906	.059	.614
Triglycerides^b^	Median (IQR)	0.03 (–0.56 to 0.27)	–0.11 (–0.32 to 0.11)	–0.12 (–0.48 to 0.30)
	*P*	.893	.265	.500
Glucose^b^	Median (IQR)	–0.20 (–0.60 to 0.50)	–0.20 (–0.70 to 0.30)	–0.15 (–0.50 to 0.30)
	*P*	.494	.349	.317
Insulin^b^	Median (IQR)	–7.65 (–15.80 to 0.60)	–0.30 (–48.20 to 15.00)	–2.00 (–35.00 to 30.30)
	*P*	.145	.801	.650
HOMA-IR^b^	Median (IQR)	–0.14 (–0.61 to –0.01)	–0.05 (–1.06 to 0.43)	–0.19 (–1.34 to 1.11)
	*P*	.098	.880	.626
HbA1c	Median (IQR)	–0.10 (–0.30 to 0.10)	0.10 (–0.20 to 0.10)	0.00 (–0.20 to 0.10)
	*P*	.277	.721	.434

*P* value was based on Wilcoxon signed-rank test for the null hypothesis of no change.

Abbreviations: ALT, ; AST, ; CAP, controlled attenuation parameter; HDL, high-density lipoprotein; HOMA-IR, Homeostatic Model Assessment–Insulin Resistance; IQR, interquartile range; LDL, low-density lipoprotein.

^a^If baseline value was unavailable, the change was computed relative to the screening or switch visit.

^b^Those with negative PCR were assigned a value of 0.

^c^Only fasting lab values were used.

At baseline, the median HOMA-IR score (IQR) was 1.60 (0.98 to 2.79), with a HOMA-IR >2.0 in 43.5% of participants (Table 3). There was no change in HOMA-IR 12 weeks post-treatment (median [IQR], 1.50 [1.00 to 2.16]). There was no effect of treatment on fasting glucose levels or HbA1c during the study period. Total cholesterol and LDL-C increased during treatment and were higher 12 weeks post-treatment compared with baseline (median change total cholesterol, 0.40 mmol/L; *P* = .02; LDL-C, 0.50 mmol/L; *P* = .001). HDL increased slightly during treatment, but there was no difference observed at 12 weeks post-treatment. There was a decrease in ApoA1 and ApoA2 at 12 weeks post-treatment compared with baseline (median change for ApoA1, –55.5 mcg/mL; *P* = .02; ApoA2 12 weeks, –29.8 mcg/mL; *P* = .047), which persisted with ApoA2 at 48 weeks (median change, –73.9; *P* = .03). There was no effect on other apolipoproteins at any time point.

At baseline, the median current health state as assessed by the EQ-5D (IQR) was 75 (70 to 85) (Table 1). Of the 5 health states assessed by this tool, Anxiety and Depression and Pain/Discomfort were the most prevalent at the start of treatment (65.2% and 82.6%, respectively). Health states remained constant during treatment and in the post-treatment period (Table 4). At baseline, >70% of participants achieved moderate to high levels of activity by IPAQ. However, there was a decrease in the activity level during the treatment period (median change [IQR], –1237 [–2994 to –120] MET; *P* = .002), which improved at 12 weeks post-treatment (median change [IQR], –336 [–1386 to 2730]; *P* = .93). AUDIT-C results were consistent with low risk of alcohol consumption and drug use at baseline and throughout the study (Tables 1 and 4).

**Table 4.** Change in Baseline Characteristics and Metabolic Parameters, Quality of Life, Alcohol Consumption, and Activity Level at Week 4, Week 12, and 12 Weeks Post-treatment

		Change Relative to Baseline^a^		
Variable		Week 4	Week 12	Week 12 Post
EQ-5D: Mobility	Median (IQR)	0.0 (0.0 to 0.0)	0.0 (0.0 to 0.0)	0.0 (0.0 to 0.0)
	*P*	1.000	.781	1.000
EQ-5D: Self-care	Median (IQR)	0.0 (0.0 to 0.0)	0.0 (0.0 to 0.0)	0.0 (0.0 to 0.0)
	*P*	.625	.750	1.000
EQ-5D: Usual Activities	Median (IQR)	0.0 (0.0 to 0.0)	0.0 (0.0 to 0.0)	0.0 (0.0 to 0.0)
	*P*	.250	.813	.531
EQ-5D: Pain/Discomfort	Median (IQR)	0.0 (–1.0 to 1.0)	0.0 (0.0 to 0.0)	0.0 (0.0 to 0.0)
	*P*	.895	.941	.984
EQ-5D: Anxiety/Depression	Median (IQR)	0.0 (0.0 to 0.0)	0.0 (0.0 to 1.0)	0.0 (0.0 to 1.0)
	*P*	.973	.364	.793
EQ-5D: Your Health Today	Median (IQR)	0.0 (–1.0 to 0.0)	0.0 (–5.0 to 4.0)	0.0 (–1.0 to 5.0)
	*P*	.716	.772	.364
AUDIT-C	Median (IQR)	0.0 (–1.0 to 0.0)	0.0 (0.0 to 1.0)	0.0 (0.0 to 2.0)
	*P*	.770	.619	.055
IPAQ-total MET-min/wk	Median (IQR)	–495.0 (–3585.0 to 279.8)	–1236.8 (–2994.0 to –120.0)	–366.0 (–1386.0 to 2730.0)
	*P*	.110	.002	.934

*P* value was based on Wilcoxon signed-rank test for the null hypothesis of no change.

Abbreviations: EQ-5D, EuroQoL Group–5 Dimensional; IPAQ, International Physical Activity Questionnaire; IQR, interquartile range.

^a^If baseline value was unavailable, the change was computed relative to the screening or switch visit.

Fifty-one adverse events were reported in 19 of 25 participants (Table 5). Two serious adverse events (psoas abscess, alcohol-related lipase elevation) were reported but were not related to study medication. One of 51 adverse events was deemed related to study medication. This was HIV RNA breakthrough, identified at the post–HCV treatment week 12 visit in a participant with a remote history of multiple-class antiretroviral resistance who was inadvertently enrolled. His HIV RNA was rapidly suppressed, with resumption of his previous, prestudy antiretroviral regimen. Drug resistance testing at the time of breakthrough revealed nucleos(t)ide and integrase resistance. No adverse event resulted in study drug dosing modification.

**Table 5.** Summary of Adverse Events in Study Participants Throughout the Study

Variable	No. (%)
Intensity	
Mild	36 (70.6)
Moderate	10 (19.6)
Severe^a^	4 (7.8)
Life-threatening	1 (2.0)
Relationship to study treatment	
Definitely	1 (2.0)
Possibly	18 (35.3)
Not related	32 (62.7)
SAE	
No	49 (96.1)
Yes^b^	2 (3.9)

Abbreviation: SAE, severe adverse event.

^a^Severe adverse events included sciatica and psoas abscess in the same patient, elevated lipase (listed as an SAE), and elevated amylase in the same patient.

^b^Study medication–unrelated episode of alcohol-related life-threatening lipase elevation (n = 1), psoas abscess (n = 1).

## DISCUSSION

As HIV treatment has evolved in recent years, there has been a remarkable increase in patients’ ability to adhere to medication, a dramatic reduction in AIDS-related morbidity and mortality, and a shift in focus to the impact of other concomitant diseases [26]. There is high prevalence of HCV infection among people with HIV [4]. Identifying safe, simple, and well-tolerated HIV and HCV treatment regimens for HIV–HCV-coinfected patients is important in preventing progression of liver disease and improving outcomes in this population. Furthermore, successful strategies that facilitate initiation and completion of HCV treatment are critical to meeting global WHO elimination targets [27].

Historically, HIV and HCV therapies were characterized by polypharmacy, high side effect profiles, and poor efficacy in the case of interferon-based HCV treatment. Previous analyses have suggested that limiting pill count and dosing frequency positively influences HIV outcomes [28, 29]. Our pilot study demonstrated that switching HIV–HCV-coinfected patients from current HIV treatment regimens to once-daily single-tablet E/C/F/TAF followed by treatment of HCV with once-daily LDV/SOF is feasible (Figure 1). Of those identified as potential candidates for this HIV–HCV treatment strategy, nearly half were screened and dosed. There were many reasons why potential candidates were not screened. Predictably, the most common reasons included prior HCV treatment, concerns identified by the patient and/or research team related to ability to adhere to the study protocol, and inability to locate the patient. The presence of detectable HIV RNA and/or HIV drug resistance history were also identified as factors that may preclude pursuit of this simplified treatment strategy. For those who were enrolled, adherence with HCV treatment was remarkably high, and side effects were minimal. There was a decline in HIV medication adherence identified in a minority of participants 12 weeks after completion of HCV treatment, which may reflect diminished verbal cues by the research team after HCV therapy and a return to individual adherence norms. Despite this, HIV RNA remained suppressed in all but 1 participant. This single case is a reminder that the presence of preexisting drug resistance must be excluded before switching to an integrase inhibitor–nucleos(t)ide regimen to minimize the chance of HIV RNA breakthrough. In this study, participants were selected based on an absence of past integrase inhibitor resistance. This is an important criterion to consider when pursuing this simplified strategy for treatment of HIV and HCV. Importantly, this regimen was safe and well tolerated, with no adverse events attributed to E/C/F/TAF or LDV/SOF. All participants dosed with LDV/SOF completed treatment and achieved SVR.

We evaluated the impact of switching to once-daily single-tablet E/C/F/TAF followed by treatment of HCV with LDV/SOF on health-related quality of life (HR-QoL), alcohol and drug use, and physical activity. Consistent with a recent Canadian HIV–HCV cohort study, DAA treatment in the HIV–HCV-coinfected population did not have any significant effect on self-perceived health states during treatment or on post-treatment response [30]. Although there was no immediate impact on HR-QoL, there was a decrease in activity level during treatment in a population that was relatively active at baseline. Further studies are needed to better understand this effect and to determine how best to support patients during this period.

In addition to evaluating the feasibility of this treatment strategy, we also conducted an intensive evaluation of the metabolic effects of the HCV treatment and cure in an HIV-coinfected population with fully suppressed HIV. HCV is recognized to influence glucose metabolism. Insulin resistance is associated with accelerated liver fibrosis [31], increased risk of hepatocellular carcinoma [32], and higher transplant complication rates [33]. Observational studies have demonstrated improved insulin sensitivity and reduced incidence of type 2 diabetes with HCV viral clearance [34–37]. In contrast to some of these studies but consistent with Messiner et al., we did not observe improvements in mean HOMA-IR with LDV/SOF treatment of HCV or after DAA dosing. This may be in part due to low prevalence of insulin resistance or impaired glucose metabolism at baseline. Furthermore, the short duration of follow-up did not allow for evaluation of the longer-term effects of HCV cure on glucose homeostasis.

HCV infection influences lipid metabolism. The HCV life cycle is dependent on the very low-density lipoprotein (VLDL) pathway. Viral replication involves the formation of complexes termed lipoviral particles [22]. Lipoviral particles are believed to facilitate binding with LDL-C receptors and are considered a mechanism by which HCV gains entry to the hepatocyte [21]. Numerous studies have demonstrated lower total cholesterol, triglycerides, and HDL-C and LDL-C levels in patients with chronic HCV infection [23, 24, 38]. Successful treatment of HCV is also associated with the reversal of hypolipidemia [39, 40]. Consistent with earlier studies, viral clearance was associated with increases in total cholesterol and LDL-C, providing further evidence that HCV induces dysregulation of lipid metabolism. The impact of increased lipids after SVR on cardiovascular disease risk is uncertain, although there is evidence that HCV cure may reduce risk [41, 42]. Nonetheless, our study highlights the need to monitor lipid levels post-treatment and reassess the need for lipid-lowering measures.

Although this exploratory study provides preliminary evidence that this 2-tablet HIV–HCV regimen is feasible and safe, there are limitations that require consideration. Given the small sample size, the potential for type I and II statistical error is acknowledged. This was a nonrandomized, open-label study, and as such it is subject to selection bias. This population was a relatively healthy and active group, with good quality of life and few comorbidities at baseline. Larger studies are needed to explore whether these results can be extrapolated to all HIV–HCV-coinfected patients. In addition, as all participants achieved SVR, it was not possible to compare outcomes between those who were cured and treatment failures.

The availability of interferon-free HCV DAAs allows for broad provision of treatment for people living with HIV–HCV. There is great potential to modify morbidity and improve outcomes in a previously challenging-to-cure population. This study provides evidence that provision of this 2-tablet daily HIV–HCV regimen is feasible, well tolerated, safe, avoids drug–drug interactions, maintains HIV suppression, and is highly curative of HCV.
